# Effects of exercise on fatigue and physical capacity in men with chronic widespread pain - a pilot study

**DOI:** 10.1186/s13102-016-0054-9

**Published:** 2016-09-06

**Authors:** Anna Ericsson, Tomas Bremell, Åsa Cider, Kaisa Mannerkorpi

**Affiliations:** 1 Institute of Neuroscience and Physiology, Department of Health and Rehabilitation/Physiotherapy, The Sahlgrenska Academy, University of Gothenburg, Gothenburg, Sweden; 2 Institute of Medicine, Department of Rheumatology and Inflammation Research, The Sahlgrenska Academy, University of Gothenburg, Gothenburg, Sweden

**Keywords:** Men, Chronic pain, Exercise, Resistance exercise

## Abstract

**Background:**

There is very limited knowledge about the effects of exercise on men with Chronic Widespread Pain (CWP), especially regarding fatigue. We wanted to investigate the effects of resistance exercise compared with pool exercise on multidimensional fatigue, psychological distress and physical capacity in men with CWP.

**Methods:**

Thirty-four men with CWP, with a mean age of 49 (SD 8, range 26–59) years, were randomised to 12 weeks of standardised pool exercise (PE) or resistance exercise (RE). The primary outcome was the Multidimensional Fatigue Inventory (MFI-20). Depression, anxiety, isometric force, pain and health-related quality of life were also assessed.

**Results:**

No significant differences were found for changes in MFI-20 between the exercise groups. The RE group improved the isometric forces of right shoulder abduction (RE: ∆2.2 SD 1.5 N, PE: ∆0.6 SD 1.2 N, *p* = 0.009), right knee flexion (RE: ∆50, SD 50 N, PE: ∆-17, SD 71 N, *p* = 0.003) and left knee flexion (RE: ∆33 SD 39, PE: ∆-9 SD 52 N, *p* = 0.017) compared with the PE group. The drop-out rate was 29 % in the RE group and 18 % in the PE group.

**Conclusions:**

Both a resistance exercise programme and a pool exercise programme improved different dimensions of fatigue in men with CWP. There were no differences in the change in fatigue over time between the exercise groups. Resistance exercise improved isometric strength compared with pool exercise. Because different types of exercise appear to improve different aspects of health, the goals could guide the choice of treatment. Further exercise studies with larger groups are needed to gain more knowledge about the effect of exercise on fatigue in men with CWP.

**Trial registration:**

ClinicalTrials.gov Identifier NCT01278641. Registration date April 2008.

## Background

Chronic widespread pain (CWP) is a common condition in primary health care. CWP has been defined as the presence of pain in the right and left side of the body, above and below the waist combined with axial pain, lasting for at least three months [1]. The prevalence of CWP in the general populations has been estimated to be between 3 % [2] and 9 % among men [3, 4] and between 6.5 % [2] and 16 % [3, 4] among women. CWP has been shown to be associated with being older, being an immigrant, being in a lower socio-economic class, having a lower educational level and having a family history of chronic pain [5]. A subcategory of patients with CWP who have more severe symptoms fulfils the criteria of fibromyalgia (FM).

Substantial fatigue is common among patients with CWP [6, 7], and fatigue has been found to be associated with pain, psychological distress, limited physical performance and decreased working capacity in patients with chronic pain [7–10]. Fatigue, pain and sleep disturbances in men with FM tend to be higher than those in healthy controls [11] but lower than those in women with FM [7, 11–14].

Aberrations in the physiological pain-processing mechanism, such as central sensitisation, interact with psychological and environmental factors in the development and maintenance of pain and tenderness in CWP and FM [15–17]. There is limited knowledge on the cause of fatigue in patients with CWP and FM; however, it has been suggested that their fatigue could be partly explained by central sensitisation as well [17, 18].

The symptoms associated with CWP and FM can be controlled to some degree with pharmacological and non-pharmacological treatments [19–21]. Different types of exercise have been shown to have positive effects on several symptoms and the physical capacity of patients with FM [22]. In addition, exercise is commonly known to enhance general health, which facilitates coping with pain, fatigue and other common symptoms of FM [23]. However, patients with chronic pain associated with aberrations in the central pain mechanisms have shown a dysfunctional response to exercise in the form of increased general pain sensitivity [24]. Patients with FM have also been found to lack pain inhibition during muscle contractions [25].

Aerobic exercise, such as pool exercise or Nordic walking, has a positive impact on pain, global well-being and physical function in women with FM and CWP [21, 26–28]. Resistance exercise has been found to improve muscle strength, overall health and current pain intensity in a recent study of women with FM [29].

Most studies of patients with CWP concern female patients only or a mix where men are in the minority, and the knowledge regarding the effects of all types of exercise on men with CWP is insufficient, especially the impact of exercise on fatigue [21, 26], which was the reason for initiating the present study.

The aim of the present study was to compare the effects of resistance exercise and pool exercise in temperate water on multidimensional fatigue, symptoms of depression and anxiety, isometric force, pain and health-related quality of life in men with CWP. The results of the present pilot study may be valuable for the planning of future exercise studies in men with CWP and FM.

## Methods

### Study design

This was a parallel randomised controlled trial that aimed to compare the effects of a 12-week pool exercise programme in temperate water with a 12-week resistance exercise programme in men with CWP. Initially, the study plan was to recruit 20 patients to each group. Due to difficulties in recruitment, only 17 patients were recruited to each group. The authors therefore decided to continue the study as a pilot study.

The primary outcome was fatigue, which was assessed in multiple dimensions with the Multidimensional Fatigue Inventory (MFI-20). The secondary outcomes were symptoms of anxiety and depression and isometric forces of shoulder abduction, knee extension, knee flexion and hand grip. The exploratory outcomes were pain intensity, the number of pain localisations and health-related quality of life. The outcomes were assessed at the study start and after 12 weeks.

### Study population

The inclusion criteria were male patients, aged 18 to 60 years, with CWP according to the American College of Rheumatology (ACR) criteria [1]. Patients who had experienced widespread pain for at least three months were classified as having CWP. The exclusion criteria were the inability to understand Swedish; severe psychiatric or somatic disorders, such as other rheumatic diseases, neurologic conditions, cancer, clinically confirmed depression or panic disorder; or having participated in resistance exercise or pool exercise at a physical therapy clinic during the preceding six months.

### Recruitment

The patients were recruited in a municipality of approximately 50 000 inhabitants in western Sweden from five primary health care centres that covered the whole municipality. Men with the diagnoses of unspecific pain or FM between 2005 and 2007 were identified by searching all patient records in the five primary health care centres and were consecutively recruited in 2008.

A total of 493 subjects in the patient records had a diagnosis of unspecific pain or FM and were thus contacted by mail with information about the randomised trial. Seventy-four of these patients replied by telephone or e-mail. Further telephone screening resulted in the exclusion of 37 patients because they did not meet the inclusion criteria (*n* = 33) or refrained from participating (*n* = 4).

**Fig. 1:**
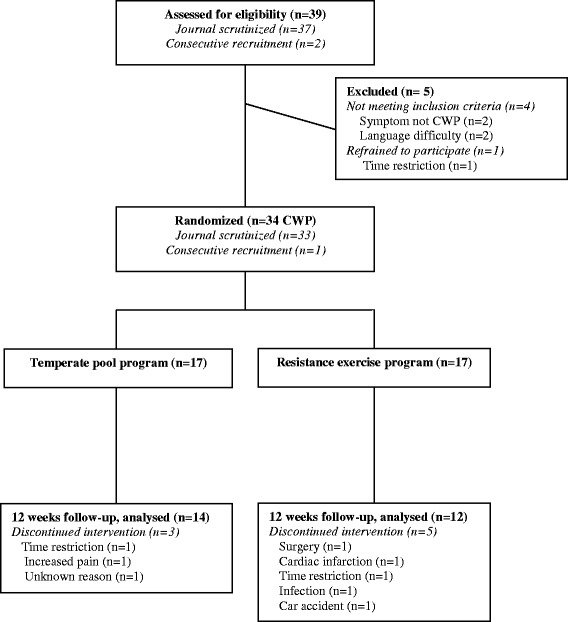
Participants’ flow

The remaining 37 patients and two additional patients with FM who were recruited consecutively were invited to the examination for the study. Five of these 39 patients were excluded because they did not meet the inclusion criteria or refrained from participating (Fig. 1).

Thirty-four patients were allocated by block randomisation to one of the two intervention group programmes. A statistician prepared sequentially numbered sealed envelopes and created the allocation sequence [30]. A person who was not involved in the examinations opened the envelopes and informed the patient about the treatment group to which he had been randomised. The final number of patients included in the randomised trial was 34, with 17 in the pool exercise group and 17 in the resistance exercise group (Fig. 1). The socio-demographic data for the two groups are given in Table 1.

**Table 1:** Demographic data of the two exercise groups

	Pool exercise (*n* = 17)	Resistance exercise (*n* = 17)
Age, years; *mean (SD)*	48 (8.7)	50 (7.5)
Duration, years; *mean(SD)*	5.0 (3.6)	5.6 (2.9)
Tender points; *mean (SD)*	7.8 (4.5)	7.4 (4.1)
Immigration; *n (%)*	5 (36)	2 (12)
Marital status; *n (%)*		
Living with adult	14 (82)	13 (76)
Not living with adult	3 (18)	4 (24)
Education, years; *n (%)*		
≤ 9	7 (41)	5 (29)
10–12	5 (29)	8 (47)
> 12	5 (29)	4 (24)
Employment; *n (%)*		
0 %	2 (12)	5 (29)
1–49 %	1 (6)	1 (6)
50–79 %	2 (12)	2 (12)
80–100 %	12 (70)	9 (53)
Sick leave; *n (%)*		
None	16 (93)	15 (88)
Part time	1 (6)	1 (6)
Full time	0 (0)	1 (6)
Disability pension; *n (%)*		
None	14 (82)	12 (70)
Part time	1 (6)	3 (18)
Full time	2 (12)	2 (12)
Medication; *n (%)*		
Analgesic/NSAID	7 (41)	12 (71)
Psychotropics	10 (59)	4 (24)
Current smoker; *n (%)*	3 (18)	5 (29)

### Procedure

#### Examinations

The examiners were trained physiotherapists who were blinded to the patients’ group assignments in the randomised trial. The patients were examined according to the ACR criteria for CWP, which included a pain localisation sheet [5] and a standardised interview. The patients completed a battery of questionnaires and performance-based tests at baseline and 12 weeks after baseline. All of the patients were instructed not to change their baseline medical treatment throughout the 12-week study period.

### Intervention

*The pool exercise programme* comprised 50-min sessions in groups of six to eight patients twice a week for a period of 12 weeks in 33 °C water, supervised by a physiotherapist. The session included aerobic exercise for endurance, strength, flexibility, coordination and relaxation.

The patients were instructed to exercise at their own rhythm and to modify the exercises individually with respect to thresholds of pain and fatigue. During the 12-week study period, they were encouraged to increase intensity and resistance with or without water equipment. The intensity during the pool exercise session was assessed with the rate of perceived exertion (RPE, 6–20) on the Borg’s scale [31] on two occasions at intervention weeks 8 and 11.

*The resistance exercise programme* was performed twice a week for 12 weeks with free weights and resistance machines in groups of approximately eight to ten patients, supervised by a physiotherapist.

The session lasted approximately one hour and included exercise for all four limbs and the back and trunk using dynamic exercises with eccentric, concentric and isometric muscle actions [32].

A standardised protocol for resistance progress was applied [32–34]. During the 12-week study period, the load was planned to be increased individually from approximately 40 % to 80 % of one repetition maximum (RM) established at baseline. One RM is defined as “the heaviest resistance that can be used for one complete repetition of an exercise” [35]. At week one, the patients performed three sets with 15–20 repetitions of each exercise. When the load increased, they still performed three sets but with fewer repetitions. All of the sessions started with ten minutes of warm-up on an ergometer bicycle.

### Background data

Information about socio-demographic data, the duration of widespread pain and pharmacological treatment was gathered in a standardised interview.

*Marital status* referred to whether the patient lived with another adult.

*Employment* referred to the percentage of full time work divided into 4 range categories. Full time work was defined as 40 h per week.

*Sick leave* and *disability pension* were divided into 3 categories: none, part time or full time sick leave or disability pension.

#### Medication

The uses of analgesics, non-steroidal anti-inflammatory drugs (NSAID) and psychotropics were considered positive if the patient used the medication regularly or as needed.

### Self-administered questionnaires

#### The MFI-20 (4-20)

The questionnaire assesses 5 subscales of fatigue: general fatigue, physical fatigue, mental fatigue, reduced motivation and reduced activity. The MFI-20 contains 20 statements that refer to aspects of fatigue experienced during the recent days. The sum score of each subscale ranges from 4 to 20 and a higher score indicates a higher degree of fatigue [36, 37]. The MFI-20 has shown satisfactory construct and content validity [38, 39] and test-retest reliability for CWP [39]. The MFI-20 has been shown to be sensitive to changes in previous exercise studies in populations with FM and CWP [27, 28, 40]; however, there is no established minimal clinically important difference for the instrument.

#### The hospital anxiety and depression scale (HADS) (0–21)

The HADS contains 14 statements, with a rating scale from zero to three, in which a higher score indicates a higher degree of distress. The scores build two subscales for anxiety (HADS-A) and depression (HADS-D), ranging from 0 to 21. A cut-off score of eight is suggested to indicate possible anxiety and depression [41]. The HADS is regarded as a valid and reliable instrument for assessing anxiety and depression in medical patients [42, 43] and has also been applied in research on CWP [27, 44].

#### FIQ pain

The subscale for pain intensity (0–100 mm) included in the Fibromyalgia Impact Questionnaire (FIQ) was applied in the study [45, 46]. The FIQ has shown good sensitivity in demonstrating therapeutic change [47], and the Swedish version of the FIQ has shown satisfactory validity and test-retest reliability for patients with FM [45].

#### Pain localisation

The localisation and distribution of pain were reported in a self-administered pain drawing with 18 predefined body regions, ranging from zero to 18, which referred to the number of body regions in pain [5].

#### Short form-36 (SF-36)

A generic instrument assessing health-related quality of life, comprising eight subscales. The subscales build two composite scores, the physical component summary (PCS) and the mental component summary (MCS) (0–100), which were both included in the study. A higher score indicates better health-related quality of life [48]. The Swedish version of the SF-36 has been validated in a general Swedish population [49].

### Performance-based tests

#### Shoulder abduction

The isometric force of shoulder abduction was measured with Isobex 3.0 (Medical Device Solution AG, Burgdorf, Switzerland) in the plane of the scapula at 45° and a shoulder elevation of 90° while in a standardised position. The subjects were seated on a chair with their feet supported by the floor. The dynamometer was placed on the floor, and the band from the dynamometer was placed proximal to the styloid process of the ulna [50].

#### Knee extension and flexion

Muscle strength of the knee extensors and flexors was measured with a pressure transducer with an amplifier (Steve Strong, Stig Starke HB, Goteborg, Sweden). The patients were seated with back support and a seat belt around the waist. Both legs hung freely with a 90° knee angle. A non-elastic strap was attached between the ankle and the pressure transducer. The subjects were instructed to pull the ankle strap in either knee extension or flexion maximally for 3 s. The best of three efforts was reported as the maximal isometric quadriceps/hamstrings force [51].

#### Hand grip

Hand grip force was measured as the sustained maximum voluntary contraction during ten seconds, measured using an electronic instrument, the Grippit [52].

### Statistics

The descriptive data are presented as the mean and standard deviation (SD) or by number and percentage. For comparisons between the exercise groups, the Mann–Whitney *U* test was used for continuous variables, and the Mantel-Haenszel chi-square test for ordinal categorical variables. For comparisons within groups, Wilcoxon’s signed rank test was used for continuous variables. To control for possible type 1 errors, the upper limit of the expected number of false significant results was calculated for the secondary and exploratory outcomes by the following formula: α/1 – α × (Number of tests – Number of significant tests), where α is the significance level. The patients who completed the 12-week examination were compared with the patients who did not complete the 12-week examination for differences in socio-demographic data, the MFI-20 subscales and the FIQ subscale for pain intensity at baseline, using the Mann–Whitney *U* test for continuous variables and the Mantel- Haenszel chi-square test for ordinal categorical variables.

## Results

### Between-group comparisons (Table 2)

**Table 2:** Baseline values, change from baseline in outcome variables, within-group and between-group differences for the pool exercise group and the resistance exercise group

	Pool exercise group			Resistance exercise group			Pool versus Resistance
	Baseline	∆ 12 weeks		Baseline	∆ 12 weeks		12 weeks
	*n* = 17	*n* = 14		*n* = 17	*n* = 12		
	Mean (SD)	Mean (SD)	*p*	Mean (SD)	Mean (SD)	*p*	*p*
Primary Outcomes							
MFI-20							
General fatigue	15.4 (2.8)	−2.0 (3.2)	0.051	16.1 (3.9)	−2.5 (3.5)	**0.040**	0.820
Physical fatigue	14.1 (4.1)	−1.9 (3.2)	**0.044**	15.2 (4.3)	−1.9 (3.4)	0.082	0.940
Mental fatigue	10.8 (4.6)	−0.6 (2.6)	0.429	12.6 (4.4)	−0.7 (2.7)	0.439	0.899
Reduced activity	11.8 (4.0)	−0.3 (3.5)	0.916	13.6 (5.1)	−1.3 (2.1)	0.065	0.446
Reduced motivation	9.6 (3.3)	−1.3 (1.7)	**0.021**	9.8 (4.1)	−0.4 (2.8)	0.561	0.176
Secondary Outcomes							
HADS							
Anxiety	8.4 (5.7)	−1.6 (2.2)	**0.028**	8.3 (5.3)	−0.8 (2.5)	0.263	0.527
Depression	5.4 (5.4)	−0.1 (2.2)	0.750	7.1 (4.0)	0.1 (2.1)	0.833	0.860
Shoulder abduction, *N*							
Right	7.5 (2.6)	0.6 (1.2)	0.059	6.5 (2.7)	2.2 (1.5)	**0.003**	**0.009**
Left	7.5 (2.5)	0.5 (1.8)	0.195	6.6 (3.1)	1.4 (2.7)	0.110	0.560
Knee extension, *N*							
Right	344.6 (99.0)	21.5 (80.4)	0.363	358.6 (108.9)	16.8 (85.5)	0.480	0.940
Left	344.2 (104.3)	36.9 (102.5)	0.245	367.1 (107.3)	30.3 (66.5)	0.170	0.742
Knee flexion, *N*							
Right	210.2 (90.2)	−17.3 (71.0)	0.551	173.4 (73.9)	49.8 (49.9)	**0.008**	**0.003**
Left	196.4 (74.9)	−8.6 (51.6)	0.851	162.4 (60.0)	33.3 (39.3)	**0.015**	**0.017**
Hand grip, *N*							
Right	354.9 (120.5)	10.2 (46.6)	0.485	371.2 (121.5)	19.3 (41.5)	0.060	0.689
Left	372.5 (107.1)	5.9 (66.8)	0.422	367.2 (118.5)	24.2 (41.9)	0.084	0.742
Exploratory Outcomes							
FIQ pain, *mm*	53.5 (28.3)	−2.5 (25.3)	0.610	69.5 (17.7)	−3.3 (13.4)	0.315	0.705
Pain localizations	8.8 (3.2)	−2.1 (2.9)	**0.022**	10.2 (3.3)	−2.3 (3.2)	**0.033**	0.860
Short Form 36							
PCS	33.8 (9.8)	4.9 (6.2)	**0.006**	36.7 (6.9)	2.2 (5.8)	0.308	0.266
MCS	46.0 (14.1)	1.9 (8.1)	0.695	35.6 (13.5)	0.5 (9.1)	0.308	0.932

*MFI-20* Multidimensional fatigue inventory, *HADS* Hospital anxiety and depression scale, *FIQ* Fibromyalgia impact questionnaire

*PCS* Physical component summary, *MCS* Mental component summary

*p*-values < 0.05 are in bold

#### Baseline

There were no significant baseline differences between the two groups of exercise in socio-demographic data or outcome variables, except for the SF-36 MCS (PE mean 46.0, SD 14.1 versus RE mean 35.6, SD 13.5; *p* = 0.029). There was also a tendency towards lower pain intensity in the pool exercise group at baseline; however, the difference between groups was not statistically significant (*p* = 0.069).

#### 12-week examination

No significant differences were found for the changes in the MFI-20 subscales at the 12-week examination between the pool exercise group and the resistance exercise group.

The isometric forces of the right arm shoulder abduction and the knee flexion in both legs improved significantly in the resistance exercise group compared with in the pool exercise group.

No statistically significant differences were found for the changes in the HADS, FIQ pain, pain localisations or SF-36 at the 12-week examination between the pool exercise group and the resistance exercise group.

#### Type 1 error

The secondary and exploratory outcomes in the between-group analyses at the 12-week examination comprised a total of 14 statistical analyses, and the upper level of the number of false significances was 0.58, which indicates that one of the significances found might be false.

### Within-group comparisons (Table 2)

#### Pool exercise group

MFI physical fatigue, MFI reduced motivation, HADS-A, the number of pain localisations and the SF-36 PCS improved significantly from baseline to the 12-week examination in the pool exercise group. A tendency towards improvement over time was also found for MFI general fatigue (*p* = 0.051).

#### Resistance exercise group

MFI general fatigue, isometric forces of the right arm shoulder abduction and knee flexion and the number of pain localisations improved significantly from baseline to the 12-week examination in the resistance exercise group.

#### Attendance

The mean attendance at the sessions for the patients who completed the 12-week examination was 82 % (SD 16) in the pool exercise group and 88 % (SD 5) in the resistance exercise group.

### Progress of exercise intensity

#### Pool exercise group

The patients’ RPE values were assessed in 4–5 patients in week 8 and week 11 to be able to illustrate the intensity of the pool exercise programme. In week 8, the RPE values ranged from 8.5 to 12 (“very light” to “fairly light”) during flexibility and coordination exercises and from 13.5 to 16 (“somewhat hard” to “very hard”) during aerobic exercise. In week 11, the patients’ RPE values ranged among the patients from 10 to 15 (“fairly light” to “somewhat hard”) during flexibility and coordination exercises from 16 to 17 (“very hard”) during aerobic exercise [31, 53].

#### Resistance exercise group

The initial load at week one was 40–50 % of 1 RM, with three sets of 15–20 repetitions. The mean increase in load from week one to week 12 was found to be between 37 % and 53 % of 1 RM for the different exercises.

### Drop-outs

Three patients in the pool exercise group were lost to follow-up at the 12-week examination (Fig. 1). In the resistance exercise group, two patients dropped out during the study period due to shoulder surgery and myocardial infarction, and three patients were lost to follow-up at the 12-week examination (Fig. 1).

There were no significant baseline differences found for age, education, employment status, sick leave, the duration of widespread pain or the number of tender points between the 26 patients who completed the 12-week examination and the eight patients who did not.

Significant baseline differences were found between the 26 patients who completed 12-week examination and the 8 patients who did not in MFI general fatigue (mean 15, SD 3 versus mean 18, SD 3; *p* = 0.006), MFI physical fatigue (mean 14, SD 4 versus mean 18, SD 2; *p* = 0.012), MFI mental fatigue (mean 11, SD 4 versus mean 15, SD 4; *p* = 0.031), MFI reduced motivation (mean 9, SD 3 versus mean 12, SD 4; *p* = 0.028), MFI reduced activity (mean 12, SD 4, versus mean 16, SD 5; *p* = 0.028) and FIQ pain (mean 56.5 mm, SD 24.5 versus mean 78.0 mm SD 17.3; *p* = 0.022).

## Discussion

The present study showed that both a 12-week supervised resistance exercise programme and a 12-week supervised pool exercise programme improved dimensions of fatigue in men with CWP. No difference was found for change in fatigue between the groups. MFI general fatigue decreased significantly over time within the resistance exercise group, and a tendency towards improvement in MFI general fatigue was found in the pool exercise group (*p* = 0.051). Physical fatigue and reduced motivation were significantly improved over time in the pool exercise group, which is in line with previous studies where the MFI-20 reduced motivation subscale has improved in groups engaging in aerobic exercise [27, 28].

The resistance exercise group improved in isometric forces of right arm shoulder abduction and knee flexion in both legs of men with CWP compared with the pool exercise group. The mean values of isometric force of the right arm shoulder abduction and knee flexion in both legs increased from baseline to post-test between 21 % and 34 % in the resistance exercise group. This result is similar to the expected improvement in resistance exercise in untrained and moderately trained healthy individuals; that is, an improvement in muscle strength of approximately 40 % and 20 %, respectively [54].

HADS-A and the physical component summary of the SF-36 improved significantly over time within the pool exercise group. The decrease in HADS-A agrees with a previous study where anxiety improved after a 20-week pool exercise programme in female patients with FM and CWP [55]. The mean difference in the SF-36 PCS between baseline and the 12-week examination was 4.3 (SD 6.1), which has been suggested to be a clinically relevant increase in chronic diseases [56–58].

No significant change in pain intensity during the intervention period was found, but both groups showed significantly fewer pain localisations after 12 weeks of exercise, which indicates an improvement in pain regardless of the type of exercise. Previous research has shown that the distribution of widespread pain tended to decrease over a period of 11 years in the general population [59]. However, because the time between examinations in the present study was only 12 weeks, the results indicate that both pool exercise and resistance exercise probably contributed to the decrease in pain spread. The pool exercise programme followed the design of a previous study of women with CWP and FM that showed a within-group improvement in multiple dimensions of fatigue and between-group improvement in MFI reduced motivation and other health-related aspects after a 20-week pool exercise period [27]. The programme provided the possibility to individually adjust the exercise to perceived pain and fatigue, and the patients appeared to increase their self-rated exertion during the 12-week study period.

The resistance exercise programme was designed in line with programmes in previous studies of resistance exercise in women with FM [33, 34] and according to recommendations for resistance exercise in healthy adults [32]. The present study included resistance exercises with free weights and machines, which are both considered to be effective for increasing strength [32]. All of the patients in the resistance exercise group did not manage to progress in their exercise according to the recommended standardised protocol [32–34] due to pain and soreness during and after exercise. However, no patient in the resistance exercise group dropped out of the study programme due to exercise-induced pain. Despite the small sample size and difficulties in increasing the exercise load for a few patients in the resistance exercise group, the present study showed significant improvements in strength, in line with previous studies of resistance exercise in women with fibromyalgia [33, 34]. Light loads that can be lifted approximately 15–25 repetitions have also been shown to increase strength in moderately trained healthy individuals [32]. Measures of isometric force are standardised, and higher values of isometric force in the back, legs and hand grip have been shown to be significantly associated with higher global health status and lower psychological distress in patients with FM [60].

There is a scarcity of studies investigating the effects of treatments on men with CWP. Thus, there are no recommendations regarding choice of exercise for men with CWP, which was the reason for conducting the present pilot study. The results of the present study can be used for the determination of sample size in future studies of men with CWP.

### Limitations

The present study was designed for 20 patients in each group, and due to difficulties with recruitment and drop-outs, only 15 versus 12 patients were analysed at the 12-week examination. This increases the risk of type 2 error because the groups in the present study might not have been sufficiently large to be able to find significant differences between the groups in terms of the change in fatigue.

Although the randomisation was performed according to protocol, the resistance exercise group showed significantly lower mental aspects of health-related quality of life and also a tendency towards higher pain intensity at baseline than the pool exercise group. This discrepancy might have caused unequal possibilities for improvement in the two groups but in favour of the pool exercise group because women with FM and CWP who have lower levels of pain intensity and psychological distress have been suggested to improve more by exercise [27].

The eight patients who dropped out appeared to report more severe fatigue and pain intensity, which might make the results less representative for patients with more severe symptoms. However, only one of the eight reported increased pain to be the reason for discontinuing the intervention. The participants in the present study were recruited by mail with information about the study. This recruitment method could have attracted persons with a positive attitude towards exercise, which might have influenced the magnitude of improvement as well as the compliance to the exercise programme. The same recruitment method was used for both groups; thus, the recruitment method would not have affected the outcome in the group comparisons.

Another limitation of the study is that one of the authors supervised the exercise sessions. However, the conditions were equal in the two groups because the same author supervised both groups. When the data were analysed, the identities of the patients were coded, which minimised the risk that the author’s possible pre-understanding would affect the interpretations of the results.

Several outcomes were included in the study as we were interested in different aspects of health. The subscales of the MFI-20 were the primary outcomes and were analysed first, followed by the secondary and exploratory outcomes. Many comparisons were carried out in the present study, and the calculation of the upper limit of the expected number of false significances indicated that one of the three significant differences in change between the groups could be false; therefore, the significance levels found should be interpreted with caution.

## Conclusions

To conclude, there were no differences between the resistance exercise programme and the pool exercise programme in fatigue; however, both groups appeared to improve in different dimensions of fatigue. The resistance exercise programme improved isometric strength compared with the pool exercise programme. The present study is a pilot study, and the results could be of guidance in the design of future studies with a larger sample, including a control group, which is needed to establish whether exercise decreases fatigue in men with CWP.

## Abbreviations

ACR: American college of rheumatology
CWP: Chronic widespread pain
FIQ: Fibromyalgia impact questionnaire
FM: Fibromyalgia
HADS: Hospital anxiety and depression scale
HADS-A: Hospital Anxiety and depression scale, subscale of anxiety
HADS-D: Hospital anxiety and depression scale, subscale of depression
MCS: Mental component summary
MFI/MFI-20: Multidimensional fatigue inventory
NSAID: Non-steroidal anti-inflammatory drugs
PCS: Physical component summary
PE: Pool exercise
RE: Resistance exercise
RM: Repetition maximum
RPE: Rating of perceived exertion
SD: Standard deviation
SF-36: Short form-36
